# Microbial trophic interactions and *mcr*A gene expression in monitoring of anaerobic digesters

**DOI:** 10.3389/fmicb.2014.00597

**Published:** 2014-11-12

**Authors:** Alejandra Alvarado, Lilia E. Montañez-Hernández, Sandra L. Palacio-Molina, Ricardo Oropeza-Navarro, Miriam P. Luévanos-Escareño, Nagamani Balagurusamy

**Affiliations:** ^1^Laboratorio de Biorremediación, Escuela de Ciencias Biológicas, Universidad Autónoma de Coahuila, TorreónMéxico; ^2^Department of Ecophysiology, Max Planck Institute for Terrestrial Microbiology, MarburgGermany; ^3^Instituto de Biotecnología – Universidad Nacional Autónoma de México, CuernavacaMéxico

**Keywords:** anaerobic digestion, microbial interactions, methanogens, *mcr*A gene expression, monitoring biodigesters

## Abstract

Anaerobic digestion (AD) is a biological process where different trophic groups of microorganisms break down biodegradable organic materials in the absence of oxygen. A wide range of AD technologies is being used to convert livestock manure, municipal and industrial wastewaters, and solid organic wastes into biogas. AD gains importance not only because of its relevance in waste treatment but also because of the recovery of carbon in the form of methane, which is a renewable energy and is used to generate electricity and heat. Despite the advances on the engineering and design of new bioreactors for AD, the microbiology component always poses challenges. Microbiology of AD processes is complicated as the efficiency of the process depends on the interactions of various trophic groups involved. Due to the complex interdependence of microbial activities for the functionality of the anaerobic bioreactors, the genetic expression of *mcr*A, which encodes a key enzyme in methane formation, is proposed as a parameter to monitor the process performance in real time. This review evaluates the current knowledge on microbial groups, their interactions, and their relationship to the performance of anaerobic biodigesters with a focus on using *mcr*A gene expression as a tool to monitor the process.

## INTRODUCTION

Anaerobic digestion (AD) is a series of unique processes that involve the reduction and oxidation of organic molecules by the complex metabolic interactions between several microbial groups. AD is widely used worldwide for the treatment of organic wastes, such as animal manures, municipal, and industrial wastewaters, and solid organic wastes such as sludge, crop, and food wastes. Carbon present in the biomass is recovered in the form of methane; a renewable form of energy and the eﬄuent can be used as organic fertilizer as it is rich in nutrients (Cho et al., 2013).

For years, several studies have focused on optimization of the design of biodigesters (Ince, 1998; Raynal et al., 1998; Bouallagui et al., 2005; Martí-Herrero, 2011), or treatment conditions (Kim et al., 2002, 2003) and the characterization and preparation of adequate waste mixtures to obtain proper C:N ratio (Hills, 1979; Yen and Brune, 2007). However, the performance and efficiency of AD depends greatly on the interactions between different active microbial groups (Talbot et al., 2008; Ali Shah et al., 2014). Therefore, characterization of the microbial community structure and the comprehension of the metabolic networks are critical to improve digestion efficiency (Shin et al., 2010). Several molecular biological analytical tools, including polymerase chain reaction (PCR) and its many variants, denaturing gradient gel electrophoresis (DGGE), fluorescence *in situ* hybridization (FISH), restriction fragment length polymorphism (RFLP) among others, have been applied in the study of the microbial communities in AD (Montero et al., 2009; Shin et al., 2010; Nelson et al., 2011; Supaphol et al., 2011).

Usually AD is conceptually divided into three or four stages, hydrolysis and/or fermentation, acetogenesis and methanogenesis. During the first stage insoluble particles of cellulose or hemicellulose contained in the substrates are hydrolyzed and converted into simple and soluble products, which are catabolized by fermentative bacteria into alcohol and fatty acids. Subsequent steps involve the oxidation of such alcohols and fatty acids with carbon chain longer than C2 to acetate by syntrophic bacteria and their activity depends on the removal of hydrogen either by CO_2_ or sulfate reduction. Finally, during methanogenesis, acetate and other methyl-containing C1 compounds are reduced to methane by aceticlastic and methylotrophic methanogens and CO_2_ is reduced by H_2_-oxidizing methanogens. Methanogens belong to the domain Archaea and are characterized by their substrate specificity, slow growth rate, and susceptibility to environmental changes, but their growth and activity is vital for the efficient functioning of AD process (Balagurusamy and Ramasamy, 1999).

Methanogenesis requieres reduction of the methyl group of methyl coenzyme M to CH_4_ by the enzyme methyl coenzyme M reductase (MCR), involving a nickel-containing factor F_430_ (Pramanik and Kim, 2013)_._ All known genomes of the methanogenic archaea encode at least one copy of the *mcr*BDCGA operon, which is composed of two alpha (*mcr*A), beta (*mcr*B), and gamma (*mcr*G) subunits (Luo et al., 2002). Moreover, all known methanogens express MCR, which catalyzes the last step in the methanogenesis (Ferry, 1999). Therefore, the presence of this enzyme is a reliable diagnostic indicator of methanogenesis in diverse environments (Reeve et al., 1997; Luton et al., 2002; Steinberg and Regan, 2009; Palacio-Molina et al., 2013). Currently various groups are involved in developing strategies to combine the analysis of differential gene expression of *mcr* alpha subunit and the traditional approaches to monitor the performance of biodigesters on real time basis. The present paper discusses the currently available knowledge on this new strategy for management of AD process.

## ANAEROBIC DIGESTION: *THE PLAYERS*

**FIGURE 1:**
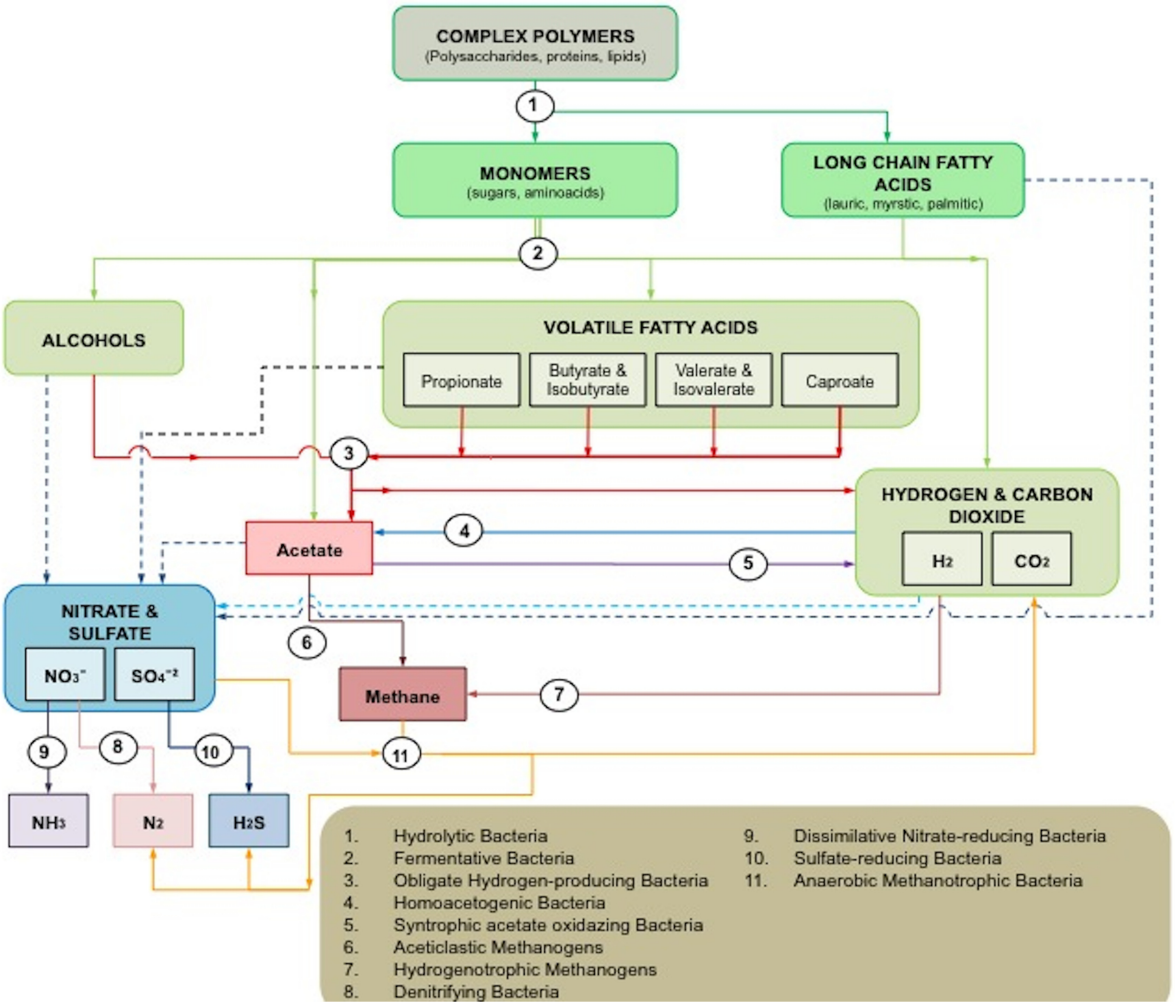
**Microbial interactions in anaerobic ecosystems.** Solid lines represent the usual pathways in anaerobic digestion. Dash lines indicate alternative pathways in which the final electron acceptors are either sulfates or nitrates.

Although many of the microorganisms involved in the process are still to be identified or cultured, at least 11 groups have been reported to interact with each other in a series of specific reactions in anaerobic ecosystems (**Figure 1**).

Hydrolytic bacteria are extremely diverse in anaerobic biodigesters, reflecting their enormous metabolic flexibility. One of the most important polysaccharides in biodigesters is cellulose, the main substrate of anaerobic cellulolytic bacteria. Cellulolytic anaerobes possess cellulosome, a multienzymatic complex, which degrades cellulose by binding to the substrate. In general, hydrolysis of polysaccharides is a slow process under anaerobic conditions. The rate and efficiency of cellulose hydrolysis in a biodigester is intrinsically related with the abundance of particulate-bound hydrolytic bacteria. In fact, the performance of a bioreactor may vary depending on the hydrolytic species that form the microbial community (Ren et al., 2007).

In one study, the microbial community was examined by DGGE and dot-blot hybridization during the start-up of two acidophilic systems at mesophilic (35∘C) and thermophilic (55∘C) conditions (Liu et al., 2002b). The reactors were fed dairy wastewater and inoculated with granular sludge. In both systems, mesophilic and thermophilic Bacteria predominated during hydrolysis, specifically the phylum Firmicutes. Actually, it is well known that in cellulolytic environments, *Clostridium* predominates (Balagurusamy, 2007). In addition, *Acetivibrio*, *Bacteroides*, *Selenomonas,* and *Ruminococcus* are some of the most common hydrolytic bacteria in the anaerobes bioreactors (Balagurusamy and Ramasamy, 1999).

In the rumen, the most similar natural environment to biodigesters, *Ruminococcus albus* and *R. flavefaciens* are the predominant gram-positive, fiber-degrading bacteria, while *Fibrobacter succinogenes* is the most abundant Gram-negative (Wanapat and Cherdthong, 2009). Commonly, hydrolytic bacteria adhere to the substrate particles along with some anaerobic fungi, which are also present in biodigesters. However, the growth of anaerobic hydrolytic fungi is slower than that of bacteria, which explains their limited presence in the community structure studies. *Neocallimastix* sp., is one the most studied anaerobic fungi in rumen, which also include *Orpimomyces, Anaeromyces, Piromyces*, and *Caecomyces* (Lynd et al., 2002; Gallert and Winter, 2005). As hydrolysis of complex compounds is catalyzed by a defined group of specific enzymes such as cellulases, proteases and lipases, this step is known as one of the most catalytically active and one that could greatly benefit from new monitoring strategies involving the analysis of gene expression profiles of key enzymes (Amani et al., 2010).

In general, hydrolysis of recalcitrant materials, such as lignin, cellulose, or hemicelluloses is a relatively slow process, and hence is often a limiting-step, normally overcome in thermophilic treatments, especially when solids contents are kept below 7%. In comparison, hydrolysis of proteins and lipids is faster (Ortega-Charleston, 2008). Proteins are generally hydrolyzed to amino acids by proteases. Microorganisms that are responsible of this reaction include species of the genera *Bacteroides, Butyrivibrio, Clostridium, Fusobacterium, Selenomonas*, and *Streptococcus* (Amani et al., 2010).

Monomeric compounds generated after hydrolysis are taken by fermentative bacteria and transformed to alcohols, volatile fatty acids (VFAs), CO_2_, or H_2_. In biodigesters, alcohols and VFAs are further transformed into the substrates for methanogenesis, namely, acetate, formate, H_2_, and CO_2_. In the presence of electron acceptors such as sulfate and nitrate, the intermediates for methanogenesis are diverted to anaerobic respiration.

Representatives of domain Bacteria are largely responsible for fermentation reactions. Among the fermentative microorganisms in the rumen are several species of *Clostridium* and *R. albus*. Meanwhile in the biodigesters fed with cow manure, members of *Clostridium, Eubacterium*, and *Bacteroides* are the abundant ones (Sivakumaran et al., 1991; Delbes et al., 2000). *Streptococcus* sp., *Lactobacillus* sp. and *Propionibacterium* are also fermentative microorganisms commonly found in the biodigesters, producing lactate or lactate and ethanol plus CO_2_ and H_2_ (Insam et al., 2010). From the phylogenetic point of view, acidogenic bacteria are widely diverse. Most of the microorganisms of this group found in biodigersters include members of the genera *Clostridium, Eubacterium*, and *Ruminococcus* (Drake et al., 2013).

The products generated by fermentation are typically VFAs, which decrease the pH and are the most common cause of failure in anaerobic systems (Chen et al., 2008; Wang et al., 2009). This acidification is a consequence of the imbalance between fermentative and syntrophic bacteria, especially obligatory hydrogen acetogenic bacteria. Although the simple monitoring of the profile of VFAs can help to prevent failures, differences between microbial populations are already very large when a decrease in the pH has been perceived (Amani et al., 2010). Hence, knowledge on the activities of microbial communities might help to anticipate this common failure even before it arises. This is probably the most important reason that microbial diversity alone is not helpful in the monitoring of anaerobic biodigesters.

Further, inhibition thresholds of VFAs vary greatly, as they depend on multiple factors, such as temperature, characteristics of feeding, source of inoculum, type of system, organic load, their state of ionization, among many others (Chen et al., 2008). But in general, acetic acid requires higher concentrations; about 2.4 g L^-1^, than the other acids to be inhibitory, and it is also a major substrate for methane production. In contrast, concentrations of propionic acid below of 900 mg L^-1^ are a sign of good performance (Wang et al., 2009).

During AD syntrophic acetogenic bacteria oxidize VFAs greater than C3 into hydrogen, acetate, and CO_2_ in association with methanogens or sulfate reducing bacteria. The oxidation of VFAs by syntrophic acetogenic bacteria is not a thermodynamically favorable process (ΔG ≥ 0). These reactions are favorable only under low partial pressure of hydrogen, which is achieved either by methanogenesis or sulfate reduction (Schink, 1997). Therefore, hydrogen metabolism is crucial in AD since at high partial pressures of hydrogen ( > 10 Pa o 10^-4^ atm) will result in accumulation of VFAs and will result in acidification of biodigesters. It has been reported that hydrogen partial pressure in biodigesters should not exceed 10^-6^ atm for the efficient oxidation of VFAs (Schink, 1997; Sieber et al., 2012).

Methane formation is the final step in AD, and it is also the most sensitive to imbalance. As a matter of fact, the amount of viable methanogens is probably the most effective indicator of a stable and effective system. Methanogenic communities are not as diverse as the others within the digesters, and they possess a specialized metabolism, characteristics that make them more likely to be inhibited. Among the seven orders of known methanogens, three are found with more frequency in the biodgesters: Methanobacteriales, Methanomicrobiales, and Methanosarcinales (Demirel and Scherer, 2008). Members of the order Methanococcales are rarely found in biodigesters; however, there is a report about the finding of these microorganisms in granular sludge treating brewery wastewater. (Liu et al., 2002a) The fifth order, Methanopyrales, includes only one hyperthemophilic species, which is unlikely to be found into anaerobic biodigesters (Bapteste et al., 2005). The recently recognized sixth order of Methanocellales contains only one genus, *Methanocella*, a hydrogenotrophic methanogen that was first isolated from a propionate-degrading culture obtained from rice paddy soils (Sakai et al., 2008). Meanwhile, the newest proposed order, Methanoplasmatales, was derived from samples of hindguts of termites and wood-feeding cockroaches. Methanoplasmatales includes members, which were previously believed to be distantly related to a different lineage in the phylum Euryarchaeota (Paul et al., 2012).

Members of Methanobacteriales, Methanococcales, and Methanomicrobiales utilize CO_2_ as electron acceptor. Hydrogen is commonly used as electron donor in this case, but some species also use formate and/or alcohols such as ethanol or isopropanol. With the exception of *Methanosphaera* (from Methanobacteriales), members of these orders cannot use acetate or one-single carbon compounds (Bonin and Boone, 2006).

Methanosarcinales are the most diverse in terms of metabolism. Acetate, hydrogen, formate, ethanol, isopropanol, and methylated compounds can be metabolized by members from this order (Kendall and Boone, 2006). *Methanosaetaceae* is the only family within Methanosarcinales that includes strictly aceticlastic anaerobes (Smith and Ingram-Smith, 2007).

In natural environments, such as swamps or rumen, populations of hydrogenotrophic methanogens are predominant, while in biodigesters; usually there are more aceticlastic methanogens (Ferry, 2010). This difference appears to be related to the amount of substrates and the presence of relatively high levels of various inhibitory compounds in the biodigesters, such as ammonia, H_2_S, and VFAs.

In general, the methanogenic pathway itself has captured the curiosity of many for decades. The pioneer work of several scientists has allowed us to know the biochemically distinctive features of methanogens. However, our understanding of how methanogenesis is coupled to energy conservation has been slower to develop (Leigh et al., 2011).

## METHANOGENESIS AND METHANOGENS

Methanogenesis usually occurs in a variety of natural anaerobic environments such as marine and freshwater sediments, rice paddies, landfill, animal digestive tracts, and hydrothermal vents. However, it has been demonstrated that these microorganisms are also able to grow in aerated places like deserts soils (Aśchenbach et al., 2013). Annually, approximately 600 million metric tons of methane is produced. Due to its potential greenhouse effect, which is 21 times higher that of CO_2_, methane emission into the atmosphere is an important concern (EPA, 2014). In this sense, AD represents an economical and effective alternative for reducing the emission of methane from organic wastes since it recovers methane as an energy source.

Methanogens can obtain energy for growth by converting a limited number of substrates to methane under anaerobic conditions. In thermodynamic terms, methanogenesis will only occur when other electron acceptors such as oxygen, nitrate, and sulfate are absent, as methanogens require a low redox potential, around -300 mv for growth and activity. Given that CO_2_ is the only electron acceptor that does not owe its abundance to photosynthesis, methanogenesis was a favored metabolism early on earth (Kasting and Siefert, 2002).

For methanogens, methane is actually a waste product. The heterodisulfide CoM-S-S-CoB formed as an intermediate in the pathway is of vital importance for the cell since its reduction is coupled to energy conservation, making the heterodisulfide the terminal electron acceptor in the respiratory chain of methanogens (Hedderich and Whitman, 2013). Methanogens use 2-mercaptoethanesulfonate (coenzyme M or CoM) as the terminal methyl carrier in methanogenesis and have four enzymes for CoM biosynthesis. Coenzyme B-Coenzyme M heterodisulfide reductase (Hdr), required for the final reaction of methanogenesis, is divided into two types, cytoplasmic HdrABC in most methanogens and membrane-bound HdrED in *Methanosarcina* species (Kaster et al., 2011).

**FIGURE 2:**
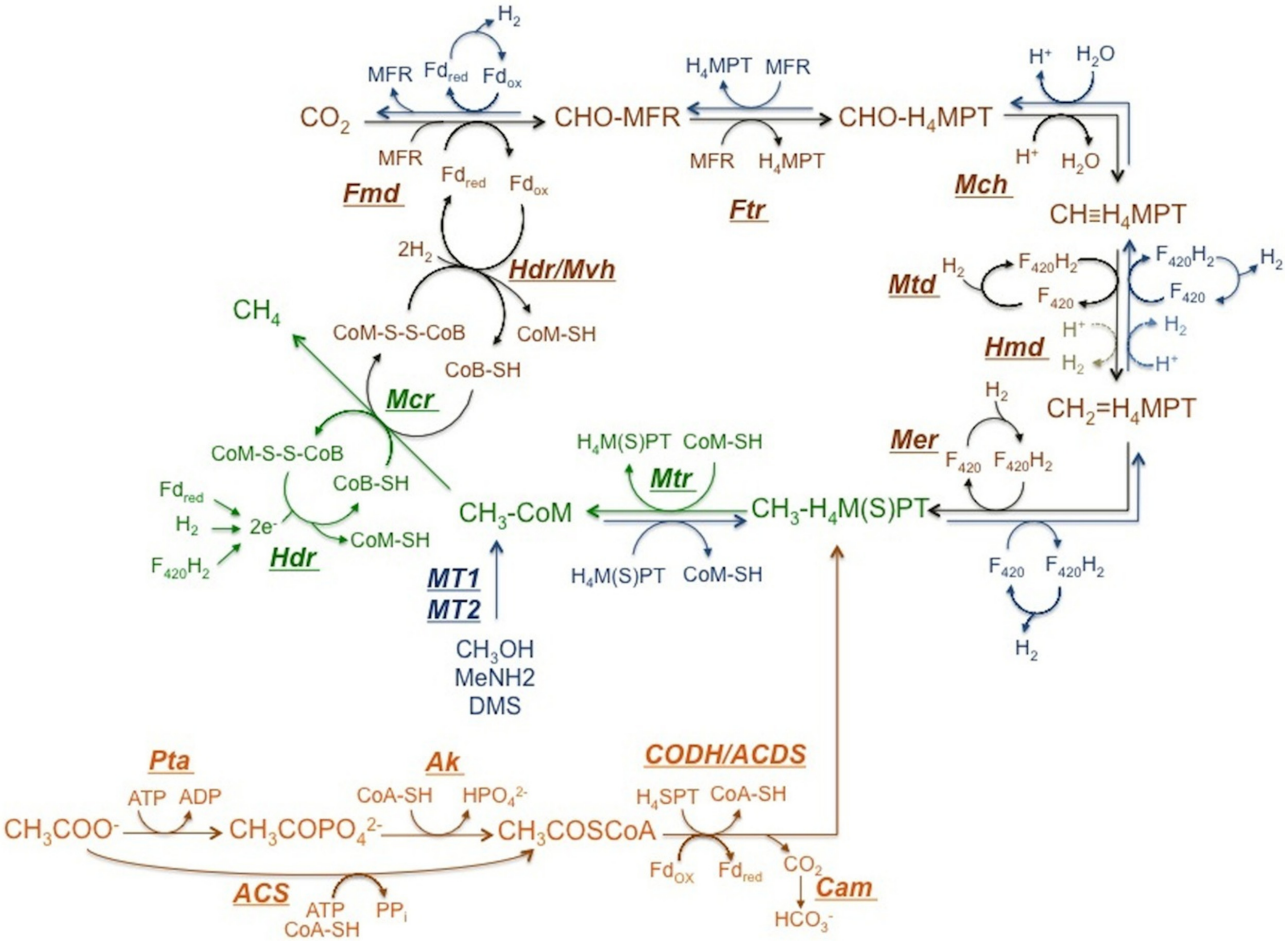
**Methanogenic Pathways.** Reactions in brown correspond to hydrogenotrophic pathway and reactions in orange belong to the aceticlastic pathway. Reactions in blue represent the pathway employed when methyl group containing C1 compounds are the substrate. Reactions in green are common in all pathways. ATP, adenosine triphosphate; H_4_SPT, tetrahydrosarcinapterin; H_4_MPT, tetrahydromethanopterin, Fd, ferredoxin; CoA-SH, coenzyme A; CoM-SH, coenzyme M; CoB-SH, coenzyme B; MFR, methanofuran; F_420_, coenzyme F_420_; Fmd, CHO-MFR dehydrogenase; Ftr, CHO-MFR:H_4_M(S)PT formyltransferase; Mch, CH≡H_4_M(S)PT cyclohydrolase; Mtd, CH_2_ = H_4_M(S)PT dehydrogenase dependent of F_420_; Hmd, CH_2_ = H_4_M(S)PT dehydrogenase independent of F_420_; Mer, CH_2_ = H_4_M(S)PT reductase; Ak, acetate kinase; Pta, phosphotransacetylase; ACS, AMP-forming acetyl-CoA synthetase; CODH/ACDS, CO dehydrogenase/acetyl-CoA synthase; Cam, carbonic anhydrase; Mtr, CH_3_-H_4_M(S)PT:CoM methyltransferase; Mcr, methyl coenzyme M reductase; Hdr, heterodisulfide reductase; Hdr/Mvh, heterodisulfide reductase/ cytoplasmic F_420_ -non-reducing hydrogenase complex.

Currently, only two types of methanogenic pathways are known, (1) methanogenesis from H_2_/CO_2_ or formate, (2) from acetate and methyl group containing C1 compounds. The conversion of methyl group to methane is common in both pathways as shown in **Figure 2** (Ferry, 2011).

In order to establish an accurate classification of methanogens, phylogenetic analysis have been made with the purpose of organize these microorganisms according to their evolutionary history (Fox et al., 1977; Bapteste et al., 2005). Initially, Bapteste et al. (2005) divided the five orders of methanogens known until that date into two major groups which they named Classes. Class I included the orders Methanobacteriales, Methanococcales and Methanopyrales and Class II comprised Methanomicrobiales and Methanosarcinales. However, it was acknowledged that Methanomicrobiales shared more traits with Class I members than with Methanosarcinales. Therefore, in 2009, an updated view for methanogens differentiation was presented. This new classification divided methanogens into three classes according to seven core methanogenesis enzymes and cofactor biosynthesis (Anderson et al., 2009). In this arrangement, Methanomicrobiales and Methanosarcinales, orders that used to be grouped in the same class, were separated into Class II and Class III, respectively, due to several unique protein signatures observed. Nevertheless, with the discovery of two novel orders, Methanocellales and Methanoplasmatales, the classification of methanogens must be updated.

On the other hand, methanogens also can be divided into two categories based on the presence or lack of cytochromes (Thauer et al., 2008). All members of Methanosarcinales possess cytochromes and methanophenazine while members of the remaining orders lack of both of them. Additionally, Methanosarcinales have the ability to grow on acetate, methanol, and H_2_/CO_2_ with a higher growth yield.

It is well documented that methanogenic communities in biodigesters are susceptible to environmental changes, especially low pH and temperature (Demirel and Scherer, 2008). However, methanogenesis in natural ecosystems is known to proceed in cold and acidic conditions that are inhospitable for biodigesters (Steinberg and Regan, 2008; Aśchenbach et al., 2013). This difference between biodigesters and natural ecosystems could be attributed to differences in the composition of the methanogenic communities (Liu and Whitman, 2008; Steinberg and Regan, 2008; Liu, 2010). Therefore, a better knowledge of these differences might lend insights into community-based strategies to increase digester stability with reduced chemical and energy inputs necessary to maintain narrow operating conditions (Steinberg and Regan, 2008).

Community studies of methanogenic population most frequently involve culture-independent techniques and molecular analysis has taken a major role in recent years. Recently biochemical markers using archaeol (2,3-diphytanyl-*O*-sn-glycerol) also have been developed (McCartney et al., 2013a). In the case of molecular analysis, various methanogen specific primers targeting 16S rRNA gene have been developed (Castro et al., 2004; Yu et al., 2005; Zhou et al., 2011). To eliminate potential problems with non-specific amplification, some researchers have developed primers for the gene sequence of the alpha subunit of the MCR, *mcr*A (Springer et al., 1995; Hales et al., 1996; Luton et al., 2002; Denman et al., 2007; Steinberg and Regan, 2008). Phylogenetic inference with *mcr*A sequence is similar to that obtained with 16S rRNA, suggesting non-lateral gene transfer. Due to the fact that methanogens may be examined exclusively from other bacteria present in the biodigesters, *mcr*A has been increasingly used for phylogenetic analysis coupled with or independently of 16S rRNA studies. Primers and methods targeting both genes for monitoring of methanogens have been also reviewed (Narihiro and Sekiguchi, 2011).

## COMPARISONS BETWEEN *mcr*A AND 16S rRNA

**FIGURE 3:**
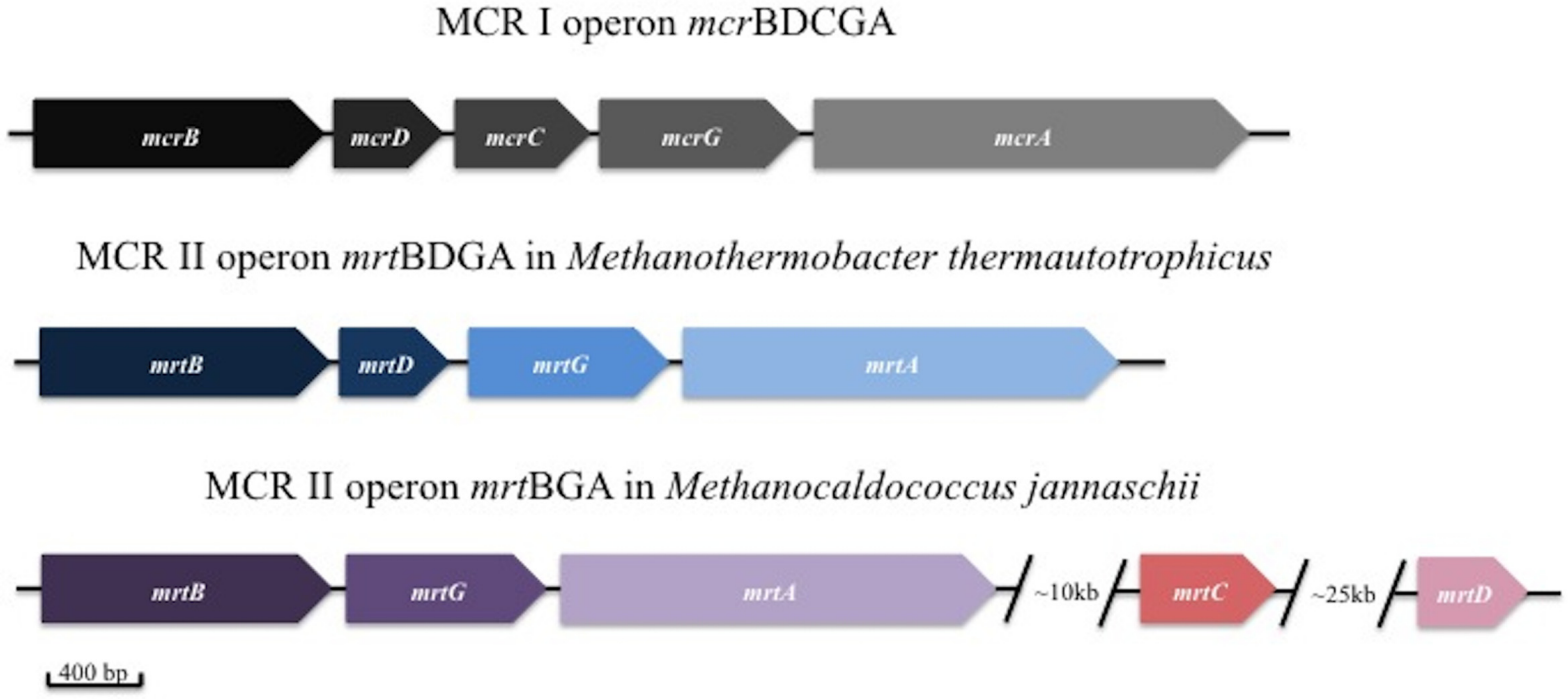
**Methyl coenzyme M reductase I (MCR I) and MCR II operon.** Structure of MCR I operon rarely varies in all methanogen species while MCR II operon is different between species containing these genes (modified from Hallam et al., 2003).

Methyl coenzyme M reductase is the unique enzyme that catalyzes the reduction of CH_3_-CoM to CH_4_ and is highly conserved in all methanogens. Two izoenzymes of MCR designated MCR I and MCR II are known and their respective operons are shown in **Figure 3**. The operon encoding MCR I, *mcr*BDCGA, prevails in all known methanogens while MCR II operon, *mrt*BDGA, is only found in some members from the orders Methanobacteriales and the Methanococcales (Garcia et al., 2000; Luton et al., 2002). Kinetic parameters are different for both isoenzymes and expression of either MCR I or MCR II seems to be dependent on hydrogen concentrations (Reeve et al., 1997). MCR II in *Methanothermobacter thermoautotrophicus (*formely known as *Methanobacterium thermoautotrophicum)* was shown to be expressed only in the earlier stages of batch cultures, then it was replaced with MCR I in late growth and stationary phases where hydrogen concentrations were lower (Pihl et al., 1994; Nölling et al., 1995).

Earlier, Rouvière and Wolfe (1987) showed that phylogenic relationships obtained with different subunits of MCR corresponded at genus level of methanogens with those derived with 16S rRNA gene. After the genes encoding for MCR subunits were available, the *mcr*A gene, which encodes the α-subunit of MCR I was selected as a phylogenetic tool for the analysis of members of the family *Methanosarcinaceae* (Springer et al., 1995). The relationships calculated with the sequences of *mcr*A and 16S rRNA showed high similarity. Subsequent studies have also confirmed that similar phylogenetic relationships can be obtained by use of 16S rRNA and *mcr*A in methanogens (Luton et al., 2002; Bapteste et al., 2005). Both strategies showed higher concurrence within the same environmental sample. However, it was also reported that 16S rRNA library of the biodigester showed less diversity than the library of *mcr*A gene (Springer et al., 1995; Steinberg and Regan, 2008).

Since then, *mcr*A gene has been established as a molecular marker for methanogenic archaea (Lueders et al., 2001) and several studies have identified the presence of *mcr*A with methanogenic activity. In 2003, environmental *mcrA* sequences were reported for the first time from a eutrophic lake (Earl et al., 2003) as well as in salt marsh sediments (Castro et al., 2004). Subsequent studies of vertebrate guts also revealed the presence of *mcrA* genes in the cow rumen (Denman et al., 2007); feces of pigs, chickens and horses (Ufnar et al., 2007a); the guts of humans (Scanlan et al., 2008; Mihajlovski et al., 2010), and the foregut of wallabies (Evans et al., 2009). A comparison between 16S rRNA and *mcr*A clone frequencies in samples of insect guts showed their strong accordance (Paul et al., 2012). As a result of this comparison arose the differentiation of a separate linage into a new order of methanogens, the Methanoplasmatales. It can be observed that use of *mcr*A gene is a potential tool in the analysis of methanogen diversity in samples from different and varied sources (Ellis et al., 2012; Lwin and Matsui, 2014).

One of the advantages of *mcr*A gene is that only one or two copies of *mcr*A have been found in sequenced methagenogens genomes, making it a more precise tool for estimating the number of these archaeas in the biodigesters than the 16S rRNA gene, which can have up to four copies per genome (Lee et al., 2009). Also, a strong correlation between *mcr*A copy number and methane production has been reported in H_2_/CO_2_- enriched cultures (Morris et al., 2014). Moreover, transcription of *mcr*A has been used to demonstrate that methanogens are metabolically active (Juottonen et al., 2008), as it is well known that these microorganisms are capable of dormancy when conditions are not optimal (Speece, 1983). Thus, identifying active members of the methanogenic population can provide a real insight into the digester performance. Likewise, because transcription is more closely related to activity, determination of *mcr*A transcript number promises to be a better indicator of good performance rather than the only *mcr*A copy number (Morris, 2011). This was proven by studies in paddy field soils, where it was discovered that under different environmental conditions, abundance of *mcr*A transcrips changed while *mcr*A gene copy number remained almost the same, suggesting that only certain members of the methanogenic community were metabolically active and responsible for methane emissions (Watanabe et al., 2009; Ma et al., 2012).

However, it was reported that quantification of gene transcript abundance in peat soils was not a reliable method since the presence of inactive and dormant cells overestimated the final values (Freitag and Prosser, 2009). Besides, MCR activity is strongly temperature dependent (Goenrich et al., 2005), and it is still unknown if post-translational modifications affect the expression of the enzyme (Kahnt et al., 2007). Therefore, the analysis of *mcr*A transcripts solely may not be efficient as a tool for monitoring biodigesters. Moreover, it should be taken into consideration that sequences of isoenzyme *mrt*A can also be part of the targets of the *mcr*A primers. Hence, overestimation of transcripts is also possible (Nettmann et al., 2008), if members of Methanobacteriales and Methanococcales are present in the samples, since *mrt*A has been observed only in both of these orders (Luton et al., 2002).

## LINKING THE *mcr*A GENE TRANSCRIPTS TO THE DYNAMICS OF THE METHANOGENIC COMMUNITY IN ANAEROBIC BIODIGESTERS

Some of the problems in using and comparing methods of *mcr*A expression studies are the choice of primers (McCartney et al., 2013b) and the differences in the PCR conditions (Steinberg and Regan, 2008). A comparison between the methods and the outcomes in each experiment requires a much more detailed analysis and for those interested on other molecular methods for environmental monitoring of methanogens, Narihiro and Sekiguchi’s (2011) review is suggested. This review discusses only the relationship between the *mcr*A gene expression and methanogenic activity, thrusting forward our knowledge on the importance of *mcr*A as a tool to monitor the functioning of the biodigester. The use of *mcr*A in different studies related to anaerobic biodigesters and natural anaerobic environments is summarized in **Table 1**.

**Table 1:** Application of *mcr*A gene in studies related to biodigester and other natural anaerobic environments.

Type of study	Primers/probes	Environment	Reference
Phylogenetic relations	MCRf/MCRr*	Pure cultures	Springer et al. (1995)
Community composition	ME1/ME2*	Blanket peat bog	Hales et al. (1996)
Community composition	MCRf/MCRr^a^ ME1/ME2^b^	Rice field soil	Lueders et al. (2001)
Community composition	ME1/ME2^b^	Oligotrophic fen	Galand et al. (2002)
Community composition	mcrAF/mcrAR*	Landfill material	Luton et al. (2002)
Community composition	ME1/ME2^b^	Lake sediments	Earl et al. (2003)
Community composition	mcrAF/mcrAR^c^	Freshwaters marshes	Castro et al. (2004)
Community composition	MCRf/MCRr^a^	Hydrothermal sediments	Dhillon et al. (2005)
Community composition	MCRf/MCRr^a^ ME1/MEr2^b^ mcrAF/mcrAR^c^	Peatland soil	Juottonen et al. (2006)
Community composition	mcrAF/mcrAR^c^ P23-2IAC*	Animal feces	Ufnar et al. (2007b)
Community composition	MeA 1046f/MeA 1435r*	Biodigester	Bauer et al. (2008)
Community composition	mcrAF/mcrAR^c^ ME1/ME2^b^	Biodigester	Nettmann et al. (2008)
Community composition	ME1/ME2^b^ MrtA_for/MrtA_rev*	Human feces	Scanlan et al. (2008)
Community composition	ME1^b^,mcrAF^c^, mlas*/ mcrA-rev*	Acidic peat bog, Biodigester	Steinberg and Regan (2008)
Community composition	MM_01_pSTC/MM_02* MM_Mbs_Fw/MM_Mbs_Rv* MMr_Mss_Fw/MMr_Mss_Rv* MM_Mx01_Fw/MM_Mx01_Rv* MM_Mx2_Fw/MM_Mx2_Rv* MM_Mx3_Fw /MM_Mx3_Rv*	Human feces	Mihajlovski et al. (2010, 2008)
Community composition	mcrAF/mcrAR^c^ ME3MF^e^/ME2^b^	Marine sediments	Merkel et al. (2010)
Community composition	mcrAF/mcrAR^c^	Biodigester	Tale et al. (2011)
Community composition	mcrAF/mcrAR^c^	Biodigester	Zhu et al. (2011)
Community composition	MCRf/MCRr^a^ ME1/MEr2^b^ mcrAF/mcrAR^c^	Biodigester	Ellis et al. (2012)
Community abundance	mlas/mcrA-rev^d^	Biodigester	Traversi et al. (2012)
Community composition	MCRf/6-FAM-MCRr^a^	Biodigester	Ma et al. (2013)
Community composition	mcrAF/mcrAR^c^	Rumen fluid	Sirohi et al. (2013)
Detection of methanogenic activity	mcrAF/mcrAR^c^	Boreal mire	Juottonen et al. (2008)
Changes in community composition	mcrAF/mcrAR^c^	Biodigester	Rastogi et al. (2008)
Changes in community composition	mcrAF/mcrAR^c^	Biodigester	Cardinali-Rezende et al. (2009)
Changes in community composition	TET-mcrAF/mcrAR^c^	Biodigester	Ács et al. (2013)
Quantification and community composition	ME1^b^/M2b* SAE716TAQ* SAR716TAQ* MCU716TAQ*	Biodigester	Shigematsu et al. (2004)
Quantification and composition of communities	mcrAF/mcrAR^c^ qmcrA-F/qmcra-R*	Rumen fluid	Denman et al. (2007)
Community composition and transcript quantification	mcrAF/mcrAR^c^ qmcrA-F/qmcra-R^f^	Rumen fluid	Guo et al. (2008)
Quantification and community composition	ME3MF*/ME2^b^	Biodigester, Marine sediments	Nunoura et al. (2008)
Quantification and composition of communities	mcrAF/mcrAR^c^ qmcrA-F/qmcrA-R^f^	Foregut of the Tammar Wallaby (*Macropus eugenii*)	Evans et al. (2009)
Quantification and community composition	mlas/mcrA-rev mbac-mcrA* mrtA* mcp* msp* MCR-7* MCR-2a* MCR-2b* fen* msar* msa*	Biodigester Acidic peat incubations	Steinberg and Regan (2009)
Quantification and changes in community composition	mlas/mcrA-rev mbac-mcrA mrtA mcp msp MCR-7 MCR-2a MCR-2b fen msar msa	Biodigester	Steinberg and Regan (2011)
Quantification and community composition	mlas/mcrA-rev^d^ msar^g^, mrtA^g^, mcp^g^ and msa^g^	Biodigester	Traversi et al. (2011)
Quantification and community composition	mcrAF/mcrAR^c^	Biodigester	Kampmann et al. (2012)
Variations in transcripts and community composition	MCRf/MCRr^a^ mlas/mcrA-rev^d^	Rice field soil	Ma et al. (2012)
Quantification and Community composition	mcrAF/mcrAR^c^ qmcrA-F/qmcrA-R^f^	Feces of horse and pony	Lwin and Matsui (2014)
Transcript and gene copy number quantification	mcrAF/mcrAR^c^	Peat soil	Freitag and Prosser (2009), Freitag et al. (2010)
Gene abundance	mcrAF/mcrAR^c^	Rumen fluid	Li et al. (2012)
Transcript and gene copy number quantification	MeA 1046f/MeA 1435r^h^	Biodigester	Munk et al. (2012)
Gene copy number quantification	mlas/mcrA-rev^d^	Cold desert soil	Aśchenbach et al. (2013)
Transcript and gene copy number quantification	qmcrA-F^f^/ mcrA-rev^d^	Biodigester Natural wetlands sediments Pure cultures	Palacio-Molina et al. (2013)
Gene copy number quantification	mcrAF/mcrAR^c^	Rice field soil	Pramanik and Kim (2013)
Variations in transcripts and community composition	mcrAF/mcrAR^c^	Rice field soil	Watanabe et al. (2009)
Variations in transcripts and community composition	MCRf/MCRr^a^	Rice field soil	Yuan et al. (2011)
Variations in transcripts and community composition	MCRf/MCRr^a^ mlas/mcrA-rev^d^	Biodigester	Zhang et al. (2014)
Transcript and gene copy number quantification, community composition	mcrAF/mcrAR^c^	Bioreactors	Morris et al. (2014)

*Primers or probes developed in same study.

*^a^Primers originally designed by Springer et al. (1995).*

*^b^Primers originally designed by Hales et al. (1996).*

*^c^Primers originally designed by Luton et al. (2002).*

*^d^Primers originally designed by Steinberg and Regan (2008).*

*^e^Primers originally designed by Nunoura et al. (2008).*

*^f^Primers originally designed by Denman et al. (2007).*

*^g^probes originally designed by Steinberg and Regan (2009).*

*^h^Primers originally designed by Bauer et al. (2008).*

## CORRELATION BETWEEN OPERATIONAL AND ENVIRONMENTAL CONDITIONS OF BIODIGESTERS AND *mcr*A

Usually, in balanced anaerobic reactors it is reported that the majority of the methanogens are aceticlastic. The effect of dilution rate and their relation to methanogenic pathways using ^13^C-labeled acetate and phylogenetic analysis of *mcrA* gene transcripts showed that transcrips of *Methanosarcina* species were the most abundant at high dilutions and that aceticlastic pathway was the major pathway for cleavage of acetate and methane production at those dilutions (Shigematsu et al., 2004). However, at low dilution rates, transcripts of *Methanoculleus* were the most abundant ones and the pathway shifted towards syntrophic acetate oxidation where hydrogenotrophic pathway was the major source for methane production. Traversi et al. (2011) reported a positive correlation between the biogas production and the presence of *Methanosarcina* and *Methanosaeta* were found in biodigesters even when most of the *mcr*A genes corresponded to members of Methanomicrobiales. It was proposed that the abundance of *Methanosarcina* was a better indicator to understand the efficiency AD process. However, other investigators have had a different experience, and the use of *Methanosarcina* species alone is not sufficient to monitor the efficiency of the biodigesters. For example, in bioreactors recovered from organic overload by addition of propionate-degrading microorganisms, *mcr*A gene copies obtained from samples of these bioreactors were associated with *Methanospirillum hungatei* and *Methanobacterium beijingense*, both hydrogenotrophic methanogens (Tale et al., 2011). The study does not report the presence of *Methanosarcina* species, and methanogenic activity is attributed to *M. hungatei* and *M. beijingense*.

Recently, analysis of *mcr*A-based libraries showed that methanogenic populations shifted substantially with modifications in substrate composition (Ács et al., 2013). Microbial community analysis of a large scale mesophilic biodigester with swine manure as substrate showed that 123 clones of *mcr*A library were assigned to 28 OTUs, of which *Methanobrevibacter* spp. (an hydrogenotrphic methanogen) was the most abundant (Zhu et al., 2011). Similarly, the predominance of hydrogenotrophic phyla (60–90%) over aceticlastic ones in six large-scale biodigesters fed with different industrial wastes has been reported (Regueiro et al., 2012). A higher predominance of hydrogenotrophic methanogens was found in a continuous anaerobic biodigester treated a mixture of fruit and meal leftovers (Cardinali-Rezende et al., 2009). Similarly, a higher proportion of OTUs clustered within the order of Methanomicrobiales for both mcr*A* and 16S rRNA libraries (79–88%) in an agricultural biogas plant fed with cattle manure and maize silage under mesophilic conditions (39∘C) was reported (Nettmann et al., 2008). Likewise, it was stated that H_2_/CO_2_ was the main substrate for methanogenesis in acidic peat (Castro et al., 2004). It was also observed that casein addition modified the population of fermenting bacteria, as well as the available hydrogen and the methanogenic community. After 5 weeks, *Methanoculleus marisnigri* increased almost twice when casein was added, and with addition of pig blood, Methanomicrobiaceae increased its abundance by 10 times. Clones related to *Methanocorpusculum parvum, Methanomassiliicoccus luminyensis*, and *Methanoculleus bourgensis* were more abundant after casein addition and decreased with pig blood. *M. luminyensis* is a methanogen that produces methane from H_2_ and methanol (Dridi et al., 2012), whereas *M. marisnigri, M. parvum*, and *M. bourgensis* as members of the Methanomicrobiales are strictly hydrogenotrophic methanogens. Similarly in another study with casein, starch and cream as substrates showed that copy numbers of *mcr*A were higher in casein fed biodigesters than the other two substrates (Kampmann et al., 2012). In the starch-fed reactor, the predominant methanogenic populations were *Methanoculleus bourgensis* and *Methanobrevibacter millerae*. These methanogens utilize H_2_ and CO_2_ for their metabolism. Similarly, the dominance of *Methanobrevibacter* and *Methanospirillum* together with uncharacterized methanogens was reported in biodigesters fed with swine manure (Zhu et al., 2011). Hydrogenotrophic methanogens, specifically of the genus *Methanoculleus* and of the order Methanomicrobiales were reported to be predominant in pulp mill wastewater treating biodigesters (Yang et al., 2013).

All these studies contradict the previously established ratio of acetate and H_2_/CO_2_ on methane production in biodgesters, 70 and 30%, respectively (Ahring, 2003). Although it is reported that stirred tank reactor conditions affected the conglomeration and structure of *Methanosarcina* and *Methanosaeta* and thereby decreased the aceticlastic activity (Kampmann et al., 2012), the predominance of hydrogenotrophic methanogens suggested that there was an increase in hydrogen concentration in these biodigesters. It is possible that the type of substrate as well as the presence and activity of syntrophic bacteria resulted in additional hydrogen and promoted hydrogenotrophic methanogenic activity.

Steinberg and Regan (2011) studied the effects of different organic loading rates on the diversity of methanogenic community in two lab-scale semi-batch reactors, one inoculated with acidic sediments and the other with anaerobic sludge. *mcr*A copies affiliated with *Methanoregula boonei* and *Methanoregula formicica* were present in the reactor with acidic sediments. *M. boonei* has been reported to show growth in acidic pH of 5 (Bräuer et al., 2006), while *M. formicica* grows at near neutral pH in syntrophic relationship with VFA oxidizers. However, *mcr*A gene copies belonging to either *Methanosarcina* species or members of the family *Methanobacteriaceae* were the dominants ones in the two biodigesters after increase in the organic loading rate (Steinberg and Regan, 2011). Likewise, an increase in *Methanosarcina* and *Methanosaeta* species was related to recovery of biodigesters performance after overloading conditions (Fernandez et al., 2000; McMahon et al., 2004; Scully et al., 2005; Hori et al., 2006). *Methanosarcina* species have been reported to grow better under high loading rates, with high acetate turnover and *Methanosaeta* species are favored in habitats with low acetate turn over (Rastogi et al., 2008). *Methanosarcina* has been defined as “the robust methanogen,” because its proven ability to tolerate the four most common causes of stress in biodigesters, *viz.*, temperature variations, organic loading rates, concentration of ammonium, and other salts (De Vrieze et al., 2012). Moreover, *Methanosarcina* has also been observed in high acidic environments, including natural wetlands (Cadillo-Quiroz et al., 2008), which suggest that this type of aceticlastic, acid-resistant methanogens might represent a good choice as inoculum for waste treatment at higher organic loads and to overcome adverse acidic pH conditions.

Palacio-Molina et al. (2013) reported that the *mcr*A gene transcription and methanogenic activity correlated to the predominant methanogenic community in one of the wetlands studied. They further reported that this correlation could not be found in samples obtained from another wetland and biodigesters. A study on the methanogenic population of a biogas plant treating cattle manure at different seasons found that *mcr*A clones related to the genus *Methanocorpusculum* were able to grow at temperatures as low as 1–5∘C, and were highly abundant in both summer (36∘C) and winter (25∘C; Rastogi et al., 2008). In contrast, clones related to *Methanosaeta concilii* were present only during summer.

Zhang et al. (2014) studied the response of methanogens to different concentrations of ammonia using *mcr*A transcripts. While T-RFLP analysis showed that members of *Methanoseataceae* were the dominant ones in all samples, the abundance of transcripts displayed variations according to the ammonia concentrations. In the case of transcripts, Methanobacteriales recorded higher number at high concentrations of ammonia. Transcripts of *Methanosarcinaceae* increased during the last stages of the experiments and this coincided with the decrease in concentration of free ammonia. In another study, addition of tea saponins recorded only 8% decrease in methane production by rumen microorganism, but decreased *mcr*A gene transcription by 76% (Guo et al., 2008). This reduction was attributed to a 79% decrease on protozoa population. It is well known that methanogens are associated with ciliates protozoa of the genera *Entodinium, Polyplastron, Epidinium*, and *Ophryoscolex* (Hook et al., 2010).

Even though *mcr*A gene is mainly employed to determine the presence and community composition of methanogens, transcriptional analysis of this gene can give us a major insight to the dynamics and performance of anaerobic digesters. Inspite of observed variations, *mcr*A gene could become an important tool for the monitoring of presence and activity in methanogens in different environments in combination with other unique biochemical properties of methanogens.

## CONCLUSION

It is a common perception and widely accepted that aceticlastic methanogens contribute nearly 70% of methane produced in animal wastes fed biodigesters. Most of the time, data on methanogenic community analysis support this idea. However, analysis of *mcr*A gene expression has broadened our knowledge on the composition and activity of methanogenic communities in biodigesters and in other anaerobic environments. It is clear that hydrogenotrophic methanogens are widely distributed, active and under some operational conditions even dominate over aceticlasic methanogens. Hence, making assumptions based only on the presence and abundance of certain methanogens groups is not a valid parameter to monitor the state of biodigesters. It can be concluded that gene expression of *mcrA* can be a potential tool in determining the active members of the methanogenic community since it gives a better insight on the metabolic dynamics within biodigesters, However, the use of *mcrA* gene expression alone or in combination with other parameters such as fluorescence as biomarkers to monitor the state of biodigester on real time basis needs further research on determining the exact of relationship between the transcripts, fluorescence, and methanogenic activity.

## AUTHOR CONTRIBUTIONS

Alejandra Alvarado, Lilia E. Montañez-Hernández, and Nagamani Balagurusamy equally contributed on the structure and manuscript of the review. All authors equally contributed on analysis, revision, and approved the final version of the manuscript. All authors agree to accountable for the accuracy and integrity of the paper.

## Conflict of Interest Statement

The authors declare that the research was conducted in the absence of any commercial or financial relationships that could be construed as a potential conflict of interest.
